# Increased Lung Expression of Anti-Angiogenic Factors in Down Syndrome: Potential Role in Abnormal Lung Vascular Growth and the Risk for Pulmonary Hypertension

**DOI:** 10.1371/journal.pone.0159005

**Published:** 2016-08-03

**Authors:** Csaba Galambos, Angela D. Minic, Douglas Bush, Dominique Nguyen, Blair Dodson, Gregory Seedorf, Steven H. Abman

**Affiliations:** 1 Departments of Pathology and Laboratory Medicine, University of Colorado School of Medicine and Children’s Hospital Colorado, Aurora, Colorado, United States of America; 2 Pediatric Surgery, University of Colorado School of Medicine and Children’s Hospital Colorado, Aurora, Colorado, United States of America; 3 Pediatrics, University of Colorado School of Medicine and Children’s Hospital Colorado, Aurora, Colorado, United States of America; 4 The Pediatric Heart Lung Center, University of Colorado School of Medicine and Children’s Hospital Colorado, Aurora, Colorado, United States of America; 5 University of Notre Dame, South Bend, Indiana, United States of America; University of Illinois College of Medicine, UNITED STATES

## Abstract

**Background and Aims:**

Infants with Down syndrome (DS) or Trisomy 21, are at high risk for developing pulmonary arterial hypertension (PAH), but mechanisms that increase susceptibility are poorly understood. Laboratory studies have shown that early disruption of angiogenesis during development impairs vascular and alveolar growth and causes PAH. Human chromosome 21 encodes known anti-angiogenic factors, including collagen18a1 (endostatin, ES), ß-amyloid peptide (BAP) and Down Syndrome Critical Region 1 (DSCR-1). Therefore, we hypothesized that fetal lungs from subjects with DS are characterized by early over-expression of anti-angiogenic factors and have abnormal lung vascular growth *in utero*.

**Methods:**

Human fetal lung tissue from DS and non-DS subjects were obtained from a biorepository. Quantitative reverse transcriptase PCR (qRT-PCR) was performed to assay 84 angiogenesis-associated genes and individual qRT-PCR was performed for ES, amyloid protein precursor (APP) and DSCR1. Western blot analysis (WBA) was used to assay lung ES, APP and DSCR-1 protein contents. Lung vessel density and wall thickness were determined by morphometric analysis.

**Results:**

The angiogenesis array identified up-regulation of three anti-angiogenic genes: *COL18A1* (ES), *COL4A3* (tumstatin) and *TIMP3* (tissue inhibitor of metallopeptidase 3) in DS lungs. Single qRT-PCR and WBA showed striking elevations of ES and APP mRNA (p = 0.022 and p = 0.001) and protein (p = 0.040 and p = 0.002; respectively). Vessel density was reduced (p = 0.041) and vessel wall thickness was increased in DS lung tissue (p = 0.033) when compared to non-DS subjects.

**Conclusions:**

We conclude that lung anti-angiogenic factors, including *COL18A1* (ES), *COL4A3*, *TIMP3* and *APP* are over-expressed and fetal lung vessel growth is decreased in subjects with DS. We speculate that increased fetal lung anti-angiogenic factor expression due to trisomy 21 impairs lung vascular growth and signaling, which impairs alveolarization and contributes to high risk for PAH during infancy.

## Introduction

Down syndrome (DS), or Trisomy 21, is associated with significant cardiovascular and pulmonary morbidity and mortality in children, including pulmonary hypertension (PAH), chronic hypoxemia, and recurrent respiratory illnesses [1–4]. Newborns with DS are at high risk of developing severe persistent pulmonary hypertension of the newborn (PPHN) shortly after birth [5] and often have more aggressive pulmonary vascular disease secondary to congenital heart disease or airways obstruction than do subjects without DS [6, 7]. However, mechanisms that increase the susceptibility of infants and children with DS to develop worse PAH and cardiorespiratory disease are poorly understood. Past studies have shown that infants dying with DS can have evidence of lung hypoplasia as demonstrated by decreased alveolarization, peripheral lung cysts, and persistence of the double-capillary network [8–10]. These early abnormalities of arrested lung development may contribute to increased susceptibility for more aggressive cardiovascular and respiratory diseases in DS, however, the genetic and molecular mechanisms responsible for abnormal lung structure and PAH in DS remain unknown.

Past studies have shown that lung vascular growth during development plays an essential role for establishing normal lung structure at birth and during early infancy [11, 12]. Experimental studies have further shown that disruption of angiogenesis during fetal life impairs alveolarization and contributes to PAH in neonatal and infant animals [11, 13, 14]. In particular, genetic or pharmacologic inhibition of vascular endothelial growth factor (VEGF) signaling reduces vascular growth, decreases alveolar formation and contributes to PAH [11, 13, 15, 16]. Whether early disruption of angiogenic signaling contributes to abnormal lung vascular and alveolar development or contributes to increased susceptibility for PAH in the setting of DS has not been previously studied.

Several anti-angiogenic factors are encoded on chromosome (Chr) 21 and their genes are triplicated in DS. These anti-angiogenic factors include endostatin (ES, encoded and cleaved from collagen 18a1, *COL18A1*), beta-amyloid protein (BAP, encoded and cleaved from amyloid-beta precursor protein, *APP*), and Down syndrome critical region 1 (DSCR1, encoded within Down syndrome critical region 1, *DSCR1*) [17]. Circulating levels of ES are increased in blood samples from children with DS [18] and ES has been shown to inhibit angiogenesis by disrupting VEGF receptor 2 (VEGFR2) signaling [19, 20]. In addition, subjects with DS are known to develop dementia and early-onset Alzheimer's disease, which is partly due to the deposition of BAP in the brain. The gene encoding the BAP precursor, *APP*, is also located on Chr 21 and BAP is present in higher concentrations in DS fetal plasma compared to controls [21]. Similar to ES, BAP also markedly inhibits angiogenesis by interfering with VEGF-VEGFR2 signaling [22]. *DSCR1*, also located on Chr 21, is overexpressed, and DS infants have high tissue levels of DSCR1 [23]. DSCR1 inhibits VEGF-induced angiogenesis by directly blocking the calcineurin-mediated signaling pathways [24]. Diminished lung VEGF expression and activity has been shown to cause lung hypoplasia in animal models and lung tissue of infants with bronchopulmonary dysplasia (BPD) [16, 25–27]. These findings support the speculation that fetuses with DS may be exposed to an “anti-angiogenic environment” *in utero*, and that these mechanisms potentially contribute to abnormal lung vascular and alveolar growth and a high risk for PAH.

Therefore, we hypothesize that chromosome 21-driven over-expression of the anti-angiogenic factors, *COL18A1*, *APP* and *DSCR1*, individually or in combination, play a previously unidentified role in disrupting lung vascular development and contribute to the pathobiology of lung hypoplasia and risk for PAH in subjects with DS. In this study, we utilized banked fetal lung tissues of DS patients from University of Maryland that were limited in number. We sought to determine whether the aforementioned anti-angiogenic genes and their proteins are over-expressed in human fetal lung tissue from subjects with DS, and whether lung vascular growth is reduced in the fetal DS lung. In addition, to identify other angiogenic genes that may contribute to abnormal lung vascular growth in DS, we also performed unbiased RNA arrays that examined multiple agents involved in angiogenic signaling. We found that *COL18A1* and *APP* mRNA and protein levels were significantly increased, while *DSCR1* mRNA and protein levels showed a trend towards increased levels in fetal DS lungs. We further report that fetal DS lungs have impaired vascular growth, including decreased microvascular density and structure. Importantly, we also found that fetal DS lungs have increased mRNA expression of two non-chromosome 21 genes with potent anti-angiogenic influence: tumstatin (*COL4A3*) and tissue inhibitor of metallopeptidase 3 (*TIMP3*). These findings support the hypothesis that increased lung expression of anti-angiogenic factors contributes to impaired lung development and may increase the risk of PAH in neonates and young infants with DS.

## Materials and Methods

### Human lung tissue

Human lung tissue obtained from the University of Maryland, Baltimore through its NICHD Brain and Tissue Bank for Developmental Disorders (NICH Contract #HHSN275200900011C, Ref. No. N01-HD-9-0011). The studies included in this report were examined by an institutional review board (IRB) of the University of Colorado School of Medicine and the Children’s Hospital Colorado and are supported through the Decedent Research Certification Program. All tissues were collected, stored and distributed while maintaining strict confidentiality, meeting appropriate HIPPA standards, with oversight provided by the University of Colorado IRB. Accordingly, limited clinical data is available. Small numbers of tissue were available for study, including 6 DS and 4 control flash frozen samples for quantitative RT-PCR, and 3 DS and 4 control formalin fixed, paraffin embedded samples were utilized for immunofluorescence, immunohistochemistry and morphometric analysis. Samples with a post mortem interval (PMI) of <9 hours were excluded from western blot analysis due to protein degradation.

### Quantitative real-time PCR (qRT-PCR)

Total RNA was extracted from human lung tissue using the RNAqueous Total RNA Isolation Kit (Life Technologies AM1912). Samples were DNase I treated with the Ambion TURBO DNA-free kit (Invitrogen AM1907) and tested for quality with an Agilent 2100 Bioanalyzer. Samples were either reverse-transcribed with RT^2^ First Strand Kit (Qiagen 330401) or with random hexamer primers and the SuperScript III First-Strand Synthesis System (Life Technologies 18080–051). These samples were amplified using either the RT^2^ Profiler PCR Array Human Angiogenesis (Qiagen PAHS-024Z) or TaqMan Gene Expression Master Mix (Life Technologies 4369016) and Taqman Assays-on-Demand probes (Life Technologies), respectively. Quantitative RT-PCR was performed on a LightCycler LC480 System (Roche). The relative gene expression was calculated using the relative standard curve method. Probes for target genes includeTaqMan Assay-on-Demand assays (Life Technologies) for *COL18A1* (Hs_00181017_m1), *APP* (Hs_00169098_m1) and *DSCR-1* (Hs_01120954). Samples assayed with the RT^2^ Profiler PCR Array Human Angiogenesis were analysed with the Qiagen online software (www.SABiosciences.com/pcrarraydataanalysis.php). Samples analysed with Taqman assays were adjusted for total RNA content by 4 housekeeping genes (Gapdh Hs_99999905_m1, GusB Hs99999908_m1, Hprt Hs_99999909_m1, and 18S Hs_99999901_S1).

### Western Blot Analysis

Proteins collected for western blot analysis were collected from whole cell lysates in RIPA buffer (Cell Signaling Technology, #9806S) with protease (Roche, catalog no. 05-892-791-001) and phosphatase inhibitors (Roche, catalog no. 04-906-845-001). Cell lysates were sonicated and centrifuged at 16,100 g for 20 minutes at 4°C. The supernatant was removed and protein content of the supernatant was determined by the bicinchoninic acid assay (Pierce Biotechnology, Rockford, IL, catalog no. 23225) with bovine serum albumin as the standard. 30 mg of protein sample per lane was resolved by SDS-polyacrylamide gel electrophoresis. Proteins from the gel were then transferred to nitrocellulose membrane and probed for with biotinilated anti-human Endostatin (R&D Systems BAF1098, 1:1000), anti-amyloid beta precursor protein (Abcam ab32136, 1:1000), anti-DSCR1 (Sigma D6694, 1:2000) and normalized to Beta-Actin (Sigma A2228, 1:10,000).

### Immunostaining and Morphometric Analysis of Fetal Lung Tissue

Histological samples were quantified for vascular density and arterial media wall thickness using the Matlab Image Processing Toolbox (Math Works, Inc. Natick, MA). Vascular density was measured by routine immunohistochemistry staining tissue for CD31 on distal vessels and a parenchyma counter background stain. Eight random images were taken from the distal lung at 20X magnification from both patient (n = 4) and control (n = 2) specimens. Vascular tissue area was identified through a threshold of the intensity of the brown staining. In the remaining image, the parenchyma tissue area was identified through a subsequent threshold. The vascular density was calculated as the ratio of microvascular area to the parenchyma area. A smooth muscle actin (SMA)-Cy3 staining with a mouse monoclonal anti-actin α smooth muscle-Cy3 (Sigma C6198-.2ml, 1:200) was used to quantify PA thickness. Random pictures of arteries were taken at 10x, 20x and 40x magnification and an average of 25 arteries/specimen were analyzed in the diseased group (n = 3) and 20 arteries/lung in the control group (n = 2). The inner and outer diameters, and cross sectional area were quantified by threshold identification of stained tissue. Arterial media wall thickness was calculated by subtracting inner diameter from outer diameter and dividing by outer diameter.

### Statistics

The basic characteristics of each group were compared using an independent two-tailed unpaired *t*-test using Microsoft Excel software. *P* values <.05 were considered as statistically significant. Quantitative RT-PCR calculations were performed in Microsoft Excel. The relative concentration of mRNA for each gene was calculated by the Roche LC480 software utilizing the relative standard curve method. The relative quantity of the gene of interest was normalized by the average relative quantity of four housekeeping genes (*Gapdh*, *GusB*, *Hprt*, and *18S*). Control and DS samples were averaged, the control samples were set to 1 and the fold-change was calculated. Western blot analysis calculations were performed in Microsoft Excel. The band intensity was calculated utilizing the Image Lab Software (BioRad). The band intensity of the protein of interest was normalized to the band intensity of β-Actin; control samples and DS samples were averaged together, the control samples were set to 1 and the fold-change was calculated.

## Results

### Abnormal alveolar and vascular structures in DS

**Fig 1:**
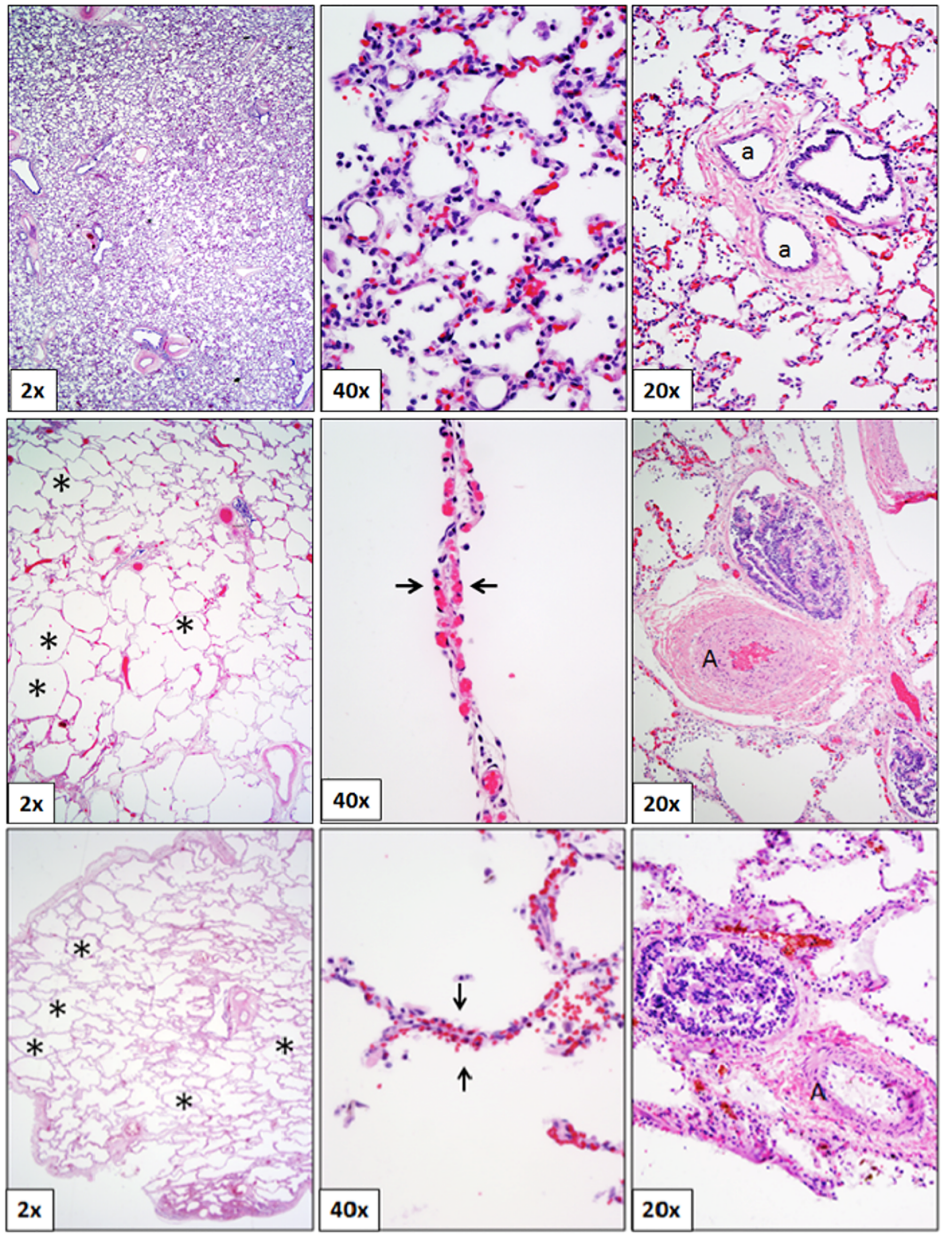
At low magnification (2x) a striking arrest of lung growth is seen in DS (middle) as characterized by decreased and enlarged alveoli in comparison with the normal lung (*alveolar spaces). At high magnification (40x), vascular developmental arrest in DS is characterized by persistence of double capillary layers (arrows) lining the alveolar spaces, which in contrast, have matured to a single layer in the control lung (top). At medium magnification (20x) arterial remodeling defect in DS lung showing a muscular pulmonary artery (A) with markedly thickened wall while intact remodeling results in thin-walled pulmonary arteries (a) in the control lung. DS lung pathologic features are strikingly similar to those of preterm infants with bronchopulmonary dysplasia (bottom).

When compared to controls, postnatal DS lung histology is characterized by diminished alveolarization, defective vascular remodeling and impaired microvascular growth (Fig 1). These features are consistent with lungs that characterize the microscopic pathology of bronchopulmonary dysplasia, a neonatal disorder with anti-angiogenic pathobiology [26].

### Expression profiles of angiogenic genes in human fetal lungs with DS

**Fig 2:**
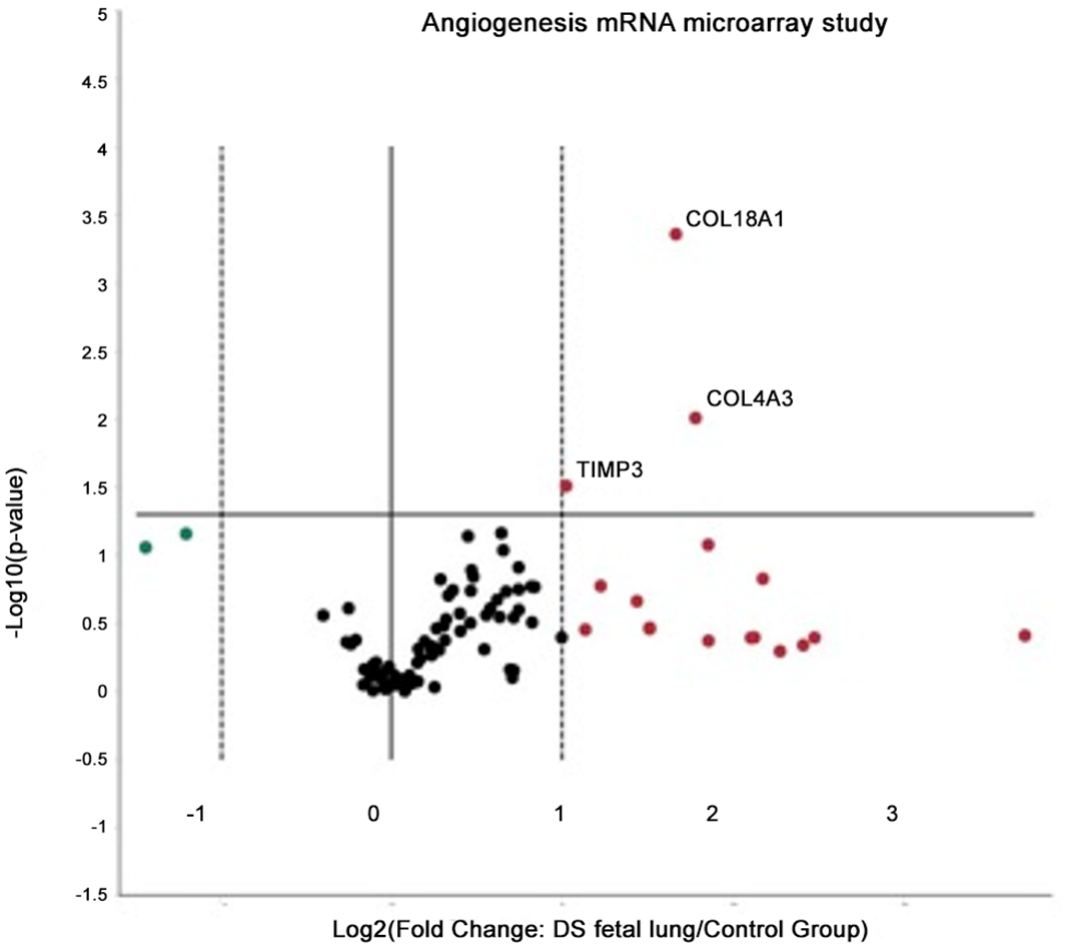
Anti-angiogenic genes are upregulated in human fetal DS lungs as shown by a volcano plot. Out of 84 human genes involved in the regulation of angiogenesis the expression of anti-angiogenic *COL18A1* (endostatin), *COL4A3* (tumstatin) and *TIMP3* (Tissue inhibitor of metallopeptidase 3) genes are significantly upregulated as measured by Human Angiogenesis RT2 ProfilerTM PCR Array (Qiagen PAHS- 024Z).

**Fig 3:**
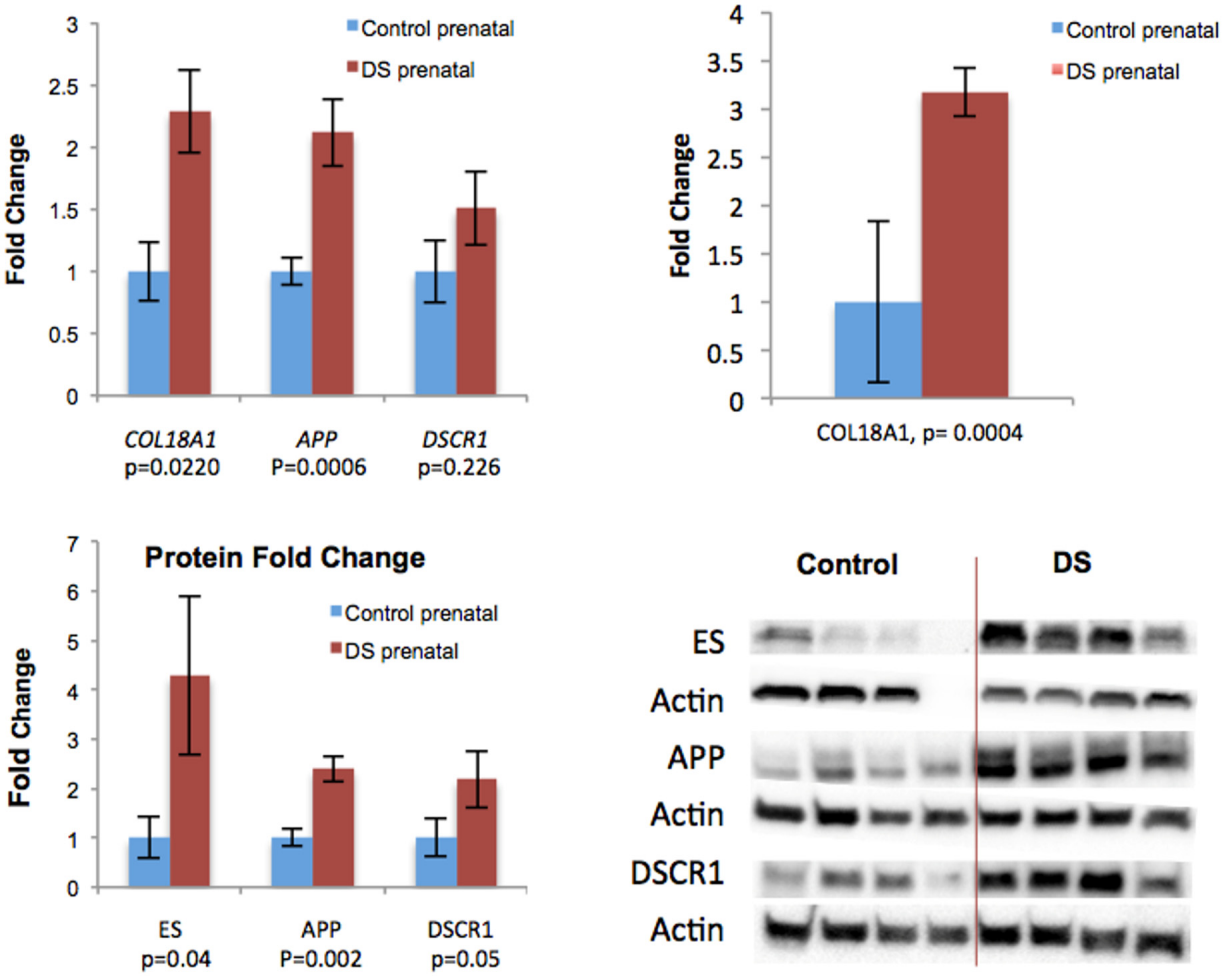
*COL18A* and *APP* and *DSCR1* mRNA expression levels are elevated in human fetal DS lungs as measured by individual qPCR (upper panel, left), while array show significant increase in *COL18A* (upper panel, right). Fetal DS lung samples show increased *COL18A*, *APP* and *DSCR1* protein expression as measured by Western blot (lower panels). While *DSCR1* mRNA expression trended towards elevation, statistical significance was noted in *COL18A* and *APP*.

Banked human fetal lung tissue with confirmed chromosomal diagnosis of DS was used to test 84 human genes that actively participate in the regulations of angiogenesis. Most notably, increased expression of the anti-angiogenic genes, *COL18A1* (endostatin), *COL4A3* (tumstatin) and *TIMP3* (tissue inhibitor of metallopeptidase 3) mRNA were noted (Fig 2, Table 1). Individual qPCR assays were used to validate findings from the qPCR human angiogenesis array data, which further confirmed a significant ES gene up regulation (p = 0.022; Fig 3).

**Table 1:** Gene expression levels of 84 genes linked to angiogenesis in fetal lung DS tissue compared to that of age-matched controls.

Symbol	Gene Name	Fold Regulation	p-value
AKT1	V-akt murine thymoma viral oncogene homolog 1	1.2188	0.151238
ANG	Angiogenin, ribonuclease, RNase A family, 5	1.18	0.468966
ANGPT1	Angiopoietin 1	-1.092	0.789725
ANGPT2	Angiopoietin 2	1.3928	0.144419
ANGPTL4	Angiopoietin-like 4	1.6411	0.704739
ANPEP	Alanyl (membrane) aminopeptidase	2.3357	0.168728
BAI1	Brain-specific angiogenesis inhibitor 1	1.6737	0.123461
CCL11	Chemokine (C-C motif) ligand 11	2.8507	0.342622
CCL2	Chemokine (C-C motif) ligand 2	1.6317	0.794725
CDH5	Cadherin 5, type 2 (vascular endothelium)	1.3215	0.361943
COL18A1	Collagen, type XVIII, alpha 1	3.1704	0.000443
COL4A3	Collagen, type IV, alpha 3 (Goodpasture antigen)	3.4335	0.009879
CTGF	Connective tissue growth factor	3.6146	0.08414
CXCL1	Chemokine (C-X-C motif) ligand 1 (melanoma growth stimulating activity, alpha)	5.3166	0.459174
CXCL10	Chemokine (C-X-C motif) ligand 10	1.6157	0.694688
CXCL5	Chemokine (C-X-C motif) ligand 5	-1.3213	0.277346
CXCL6	Chemokine (C-X-C motif) ligand 6 (granulocyte chemotactic protein 2)	-1.1915	0.246302
CXCL9	Chemokine (C-X-C motif) ligand 9	5.568	0.40399
EDN1	Endothelin 1	1.2125	0.497121
EFNA1	Ephrin-A1	1.2582	0.198187
EFNB2	Ephrin-B2	-1.0789	0.986623
EGF	Epidermal growth factor	-1.0119	0.660505
ENG	Endoglin	1.3784	0.18275
EPHB4	EPH receptor B4	1.468	0.275991
ERBB2	V-erb-b2 erythroblastic leukemia viral oncogene homolog 2, neuro/glioblastoma derived oncogene homolog (avian)	-1.0272	0.964298
F3	Coagulation factor III (thromboplastin, tissue factor)	4.5148	0.14954
FGF1	Fibroblast growth factor 1 (acidic)	1.1138	0.489156
FGF2	Fibroblast growth factor 2 (basic)	-1.0367	0.848569
FGFR3	Fibroblast growth factor receptor 3	1.0231	0.866841
FIGF	C-fos induced growth factor (vascular endothelial growth factor D)	1.176	0.543677
FLT1	Fms-related tyrosine kinase 1 (vascular endothelial growth factor/vascular permeability factor receptor)	1.763	0.170323
FN1	Fibronectin 1	-1.2019	0.435581
HGF	Hepatocyte growth factor (hepapoietin A; scatter factor)	1.0752	0.764985
HIF1A	Hypoxia inducible factor 1, alpha subunit (basic helix-loop-helix transcription factor)	-1.0409	0.74062
HPSE	Heparanase	1.7865	0.172001
ID1	Inhibitor of DNA binding 1, dominant negative helix-loop-helix protein	1.5907	0.185447
IFNA1	Interferon, alpha 1	1.9938	0.403034
IFNG	Interferon, gamma	2.7063	0.218112
IGF1	Insulin-like growth factor 1 (somatomedin C)	-1.1221	0.894235
IL1B	Interleukin 1, beta	4.301	0.405674
IL6	Interleukin 6 (interferon, beta 2)	3.6188	0.426937
IL8	Interleukin 8	4.8389	0.508309
ITGAV	Integrin, alpha V (vitronectin receptor, alpha polypeptide, antigen CD51)	-1.0154	0.935105
ITGB3	Integrin, beta 3 (platelet glycoprotein IIIa, antigen CD61)	1.2409	0.422382
JAG1	Jagged 1	1.2816	0.181148
KDR	Kinase insert domain receptor (a type III receptor tyrosine kinase)	1.3184	0.268971
LECT1	Leukocyte cell derived chemotaxin 1	-2.717	0.087986
LEP	Leptin	1.1093	0.617263
MDK	Midkine (neurite growth-promoting factor 2)	-2.3059	0.069812
MMP14	Matrix metallopeptidase 14 (membrane-inserted)	1.3626	0.072875
MMP2	Matrix metallopeptidase 2 (gelatinase A, 72kDa gelatinase, 72kDa type IV collagenase)	1.0344	0.870017
MMP9	Matrix metallopeptidase 9 (gelatinase B, 92kDa gelatinase, 92kDa type IV collagenase)	2.1944	0.353563
NOS3	Nitric oxide synthase 3 (endothelial cell)	1.6746	0.252676
NOTCH4	Notch 4	1.5499	0.285089
NRP1	Neuropilin 1	1.0102	0.901356
NRP2	Neuropilin 2	1.1993	0.346282
PDGFA	Platelet-derived growth factor alpha polypeptide	1.3824	0.129482
PECAM1	Platelet/endothelial cell adhesion molecule	1.2351	0.330842
PF4	Platelet factor 4	1.1431	0.429132
PGF	Placental growth factor	1.084	0.879074
PLAU	Plasminogen activator, urokinase	1.6746	0.178779
PLG	Plasminogen	1.4919	0.246584
PROK2	Prokineticin 2	1.6317	0.699961
PTGS1	Prostaglandin-endoperoxide synthase 1 (prostaglandin G/H synthase and cyclooxygenase)	1.1235	0.573455
S1PR1	Sphingosine-1-phosphate receptor 1	1.0921	0.858494
SERPINE1	Serpin peptidase inhibitor, clade E (nexin, plasminogen activator inhibitor type 1), member 1	4.3585	0.403281
SERPINF1	Serpin peptidase inhibitor, clade F (alpha-2 antiplasmin, pigment epithelium derived factor), member 1	1.5607	0.069171
SPHK1	Sphingosine kinase 1	1.6402	0.287893
TEK	TEK tyrosine kinase, endothelial	1.2466	0.296592
TGFA	Transforming growth factor, alpha	1.7681	0.311782
TGFB1	Transforming growth factor, beta 1	1.5339	0.212862
TGFB2	Transforming growth factor, beta 2	1.0543	0.99407
TGFBR1	Transforming growth factor, beta receptor 1	1.0114	0.768133
THBS1	Thrombospondin 1	-1.1182	0.689432
THBS2	Thrombospondin 2	1.1896	0.931982
TIE1	Tyrosine kinase with immunoglobulin-like and EGF-like domains 1	1.376	0.315951
TIMP1	TIMP metallopeptidase inhibitor 1	1.4562	0.492715
TIMP2	TIMP metallopeptidase inhibitor 2	1.1753	0.492226
TIMP3	TIMP metallopeptidase inhibitor 3	2.0298	0.031061
TNF	Tumor necrosis factor	13.0834	0.389879
TYMP	Thymidine phosphorylase	2.8491	0.347873
VEGFA	Vascular endothelial growth factor A	1.1119	0.845966
VEGFB	Vascular endothelial growth factor B	1.0392	0.814579
VEGFC	Vascular endothelial growth factor C	-1.0683	0.776789
ACTB	Actin, beta	-1.0658	0.613041
B2M	Beta-2-microglobulin	1.5724	0.092433
GAPDH	Glyceraldehyde-3-phosphate dehydrogenase	-1.1576	0.420154
HPRT1	Hypoxanthine phosphoribosyltransferase 1	-1.0789	0.640437
RPLP0	Ribosomal protein, large, P0	-1.1813	0.451793
HGDC	Human Genomic DNA Contamination	1.9938	0.403034

### *COL18A1*, *APP* and *DSCR1* mRNA and protein are overexpressed in human DS fetal lungs

In addition to the array studies, individual qPCR and western blot assays were performed to specifically determine whether *COL18A1*, *APP* and *DSCR1* mRNA and proteins (S1–S9 Figs) are elevated in human fetal DS lungs. We found significantly high expression of *COL18A1* and *APP* mRNA (p = 0.022 and p = 0.001 respectively) as well as ES and APP protein expression (p = 0.040 and p = 0.002, respectively) in fetal DS lungs when compared to that of age-matched controls (Fig 3). The *DSCR1* mRNA and protein expression trended toward overexpression in DS lungs, and only reached significance at protein level (P = 0.226, P = 0.05, respectively, (Fig 3).

### Decreased vessel density and increased wall thickness in DS fetal lungs

**Fig 4:**
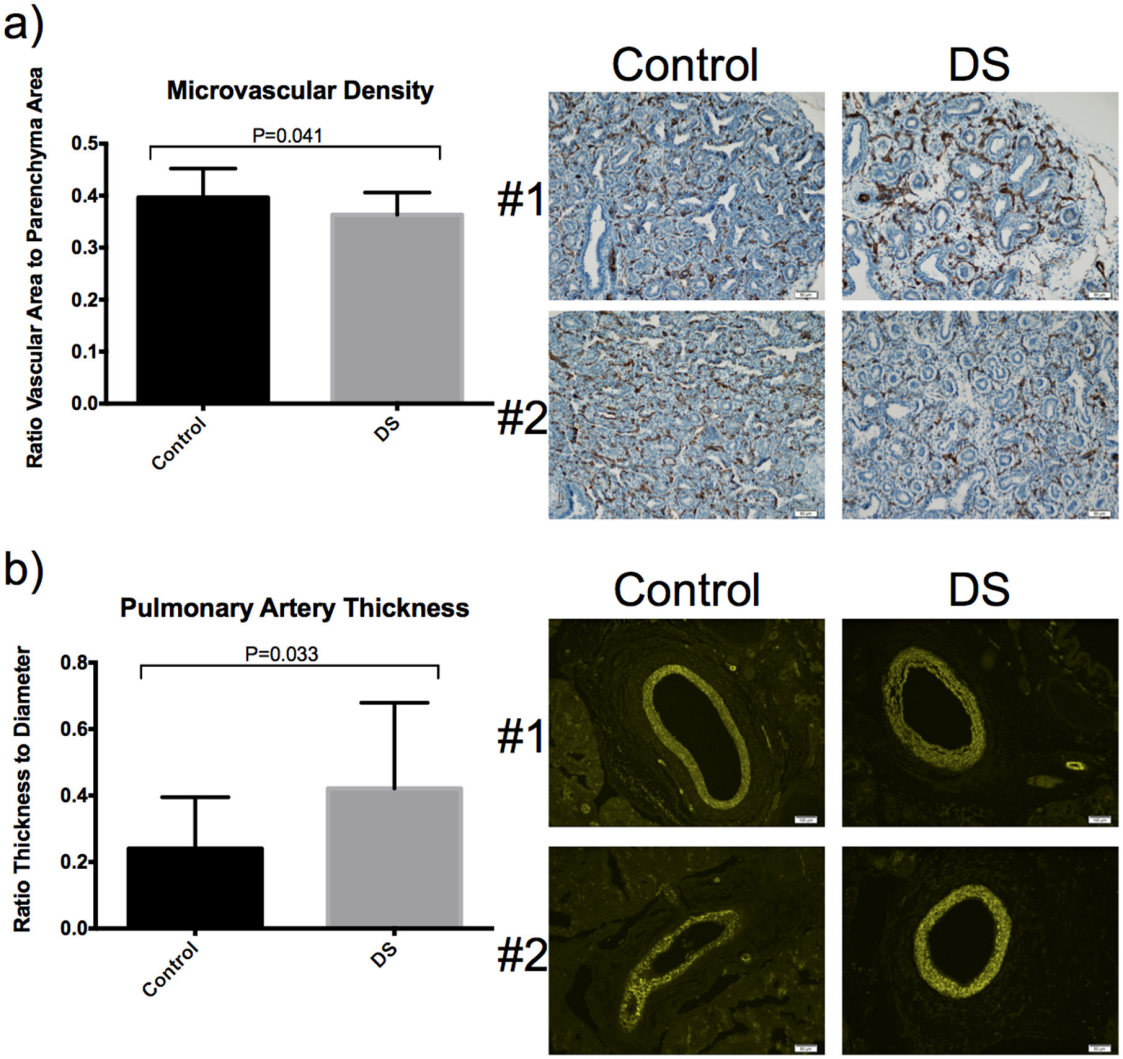
Vascular growth is impaired in human fetal DS lungs. A. Peripheral microvascular density (highlighted by CD31 immunostain) is significantly decreased in fetal DS lungs when compared to that of control lungs measured by MathLab Image Processing Toolbox Computer Program. B. Pulmonary arterial wall thickness in prenatal DS lungs is significantly increased when compared to control lungs measured by MathLab Image Processing Toolbox Computer Program.

Vessel density, as measured by computer based image analysis software, was significantly lower in fetal DS lung tissue when compared to controls (p = 0.041; Fig 4, upper panel). We also found that vascular wall thickness was increased in the DS group (p = 0.033; Fig 4, lower panel).

## Discussion

In this study we suggest that impaired lung development in Down syndrome (DS) is caused by increased anti-angiogenic activity during *in utero* lung development. Utilizing DS fetal lung tissue, we demonstrate diminished microvascular density and increased pulmonary arterial vascular thickness (Fig 4) when compared to non-DS controls, suggesting impaired vascular development in DS. We report elevated lung tissue specific mRNA for anti-angiogenic factors in DS including Collagen18a1 (*COL18A1)*, amyloid protein precursor (*APP*), tumstatin (*COL4A3)* and tissue inhibitor of metallopeptidase 3 (*TIMP3)*(Figs 2 and 3). Elevated lung tissue specific protein for endostatin (ES) and APP were additionally noted in fetal DS lungs compared to controls (Fig 3). Finally, mRNA and protein for Down syndrome critical region 1 (DSCR-1) were elevated in DS fetal lungs; significant for protein levels (Fig 3). These findings suggest an *in utero* anti-angiogenic milieu may contribute to the pulmonary vascular phenotype typical in patients with Down syndrome.

The strikingly under-developed pulmonary vascular and alveolar phenotype in patients with Down syndrome (DS) is similar to the lungs of infants with bronchopulmonary dysplasia (Fig 1) and likely contributes to the increased incidence of pulmonary hypertension in this patient population. These findings are consistent regardless of gestational age, suggesting Down syndrome specific pathomechanisms for disordered lung development. In this study, we are the first to report elevation of anti-angiogenic factors in fetal DS lung tissue including chromosome 21 specific (ES, APP, DSCR-1) and non-chromosome 21 specific factors (tumstatin and TIMP3). *In utero* inhibition of pulmonary vascular growth through these potent inhibitors of VEGF activity likely contributes to the disordered micro-vascular and alveolar growth during critical periods of fetal lung development. These findings suggest that cardiopulmonary diseases in Down syndrome may primarily be disorders of disrupted vascular development.

Prior angiogenesis studies in DS have focused on the beneficial effects of elevated anti-angiogenic factors in preventing vascular lesions and solid tumors [17, 28]. Here we emphasize the potential detrimental effects of an elevated anti-angiogenic environment during critical periods of *in utero* lung development. Although it has been long recognized that infants with DS have a markedly increased risk for developing respiratory disease and severe pulmonary hypertension (PAH), underlying mechanisms that contribute to respiratory disease are poorly understood. Previous reports of elevated serum ES, BAP and tissue DSCR1 [18, 21, 23] have been identified in patients with DS, however the current research emphasis has focused on neoplastic disorders and neurologic dysfunction. Our findings provide evidence that DS lungs carry features of disrupted pulmonary angiogenesis in fetal life with evidence of defective pulmonary vascular remodeling similar to that seen in patients with pulmonary arterial hypertension. Of note, a recent study implicated increased circulating serum ES as a potential biomarker to predict adverse outcomes in non-DS adult patients with PAH [29]. Our findings of elevated ES mRNA and protein in the DS fetal lung may implicate ES and other anti-angiogenic proteins as key factors contributing to the early development of PAH in patients with DS.

It is very likely that overexpressed anti-angiogenic molecules synergistically inhibit DS lung vascular development. For example the function of *COL18A1* and *COL4A3* are likely symbiotic as *COL18A1* blocks VEGF induced endothelial cell migration but not proliferation, while *COL4A3* inhibits VEGF induced proliferation and not migration [30]. In addition to ES and BAP, TIMP3 has been shown to block the VEGF-VEGFR2 binding site further inhibiting VEGF signaling [31]. Moreover, ES may enhance the anti-angiogenic action of TIMP3 as ES has been shown to inhibit certain matrix metalloproteinases (MMP), reducing extracellular matrix degradation and blocking vessel growth [32]. While *TIMP3* and *COL4A3* are not expressed on human Chr 21, their mRNA overexpression in fetal DS lungs may be related to an as yet described abnormality in remodeling of extracellular matrix proteins in patients with DS. TIMP3 is known to upregulate matrix metalloproteinase-9 (MMP-9) a proteinase that releases tumstatin from the extracellular matrix [33]. Further investigation may focus on human Chr 21 related *TIMP3* modulators of gene or protein expression. In addition to the candidate genes included in this study, there are two additional known potent anti-angiogenic factors on the 21^st^ human Chr. Over expressed dual-specificity tyrosine-(Y)-phosphorylation regulated kinase 1A (*DYRK1A*) and A disintegrin and metalloproteinase with thrombospondin motifs 1 (*ADAMTS1*) may contribute to the global anti-angiogenic milieu in the DS lung [34, 35]. Interestingly, several of these anti-angiogenic factors, in addition to BAP, play a critical role in the development of neurodegenerative disorders that characterize DS [35].

The individual and synergistic effects of anti-angiogenic factors likely play a significant role in increasing the risk of developing pulmonary vascular disease in patients with DS. The common comorbid conditions in DS including increased pulmonary vascular hemodynamic stresses from reduced vessel density and structural cardiac defects as well as chronic hypoxemia from airways disease and obstructive sleep apnea likely contribute to the accelerated development of PAH [1, 7, 8, 36, 37]. While ES has been directly correlated with PAH disease severity in non-DS adults with PAH [29], a direct correlation between elevated anti-angiogenic factors and the development of PAH will require further investigation.

This novel investigation links the human Chr 21 related anti-angiogenic milieu to the pulmonary vascular phenotype in patients with DS. While the role of anti-angiogenic factors have been studied with respect to inhibition of tumor growth and neurologic development in this unique patient population, the effect of upregulated anti-angiogenic factors and lung development in patients with DS has never been studied. This study is limited by its small sample size and likely because of selective tissue degradation we were only able to obtain ES and APP but not BAP protein levels. Further, ontogeny has not been characterized for *COL18A1*, *APP* and *DSCR1* genes and postnatal gene and protein expression into childhood remains unknown, both of which we plan to study in the future. Identifying the cellular source of the antiangiogenic factors as well as quantification of pulmonary arterial endothelial and smooth muscle cells is of future importance.

In summary, utilizing human tissues, we established that potent Chr 21 related anti-angiogenic factors are significantly overexpressed in human DS fetal lung. By showing that fetal human lung has diminished angiogenesis we speculate that in utero active anti-angiogenic mechanisms significantly contribute to lung hypoplasia and to the increased risk of PAH in DS. Because of elevated anti-angiogenic gene dosage of triplicated Chr 21, DS has been viewed as a syndrome that carries strong protection against angiogenic diseases. Our data suggest that the pathologic effect of anti-angiogenic function should also be considered in this patient population. We detected abnormal *in utero* vessel growth in DS lungs and we propose that this may also take place in other organs including the developing brain. If proven, our findings could serve as basis for translational approaches that focus on early intervention emphasizing angiogenic targets with the goal of reducing pulmonary and neurodegenerative morbidity and mortality in neonates and children with DS.

## Supporting Information

S1 Fig APP protein expression. Western blot gel probed with amyloid protein precursor (APP).
(TIF)

S2 Fig Actin protein expression. Western blot APP gel, probed with actin endogenous control.
(TIF)

S3 Fig APP protein blot. White light image of APP western blot.
(TIF)

S4 Fig DSCR1 protein expression. Western blot gel for Down syndrome critical region 1 (DSCR1).
(TIF)

S5 Fig Actin protein expression. Western blot gel, actin control for DSCR1.
(TIF)

S6 Fig DSCR1 protein blot. White light image of DSCR1 western blot.
(TIF)

S7 Fig ES protein expression. Western blot gel for endostatin (ES).
(TIF)

S8 Fig Actin protein expression. Western blot gel, actin control for ES.
(TIF)

S9 Fig ES protein blot. White light image of endostatin western blot.
(TIF)

## Abbreviations

APP: amyloid-beta precursor protein
BAP: beta-amyloid protein
Chr: chromosome
DSCR1: Down syndrome critical region-1
DS: Down syndrome
ES: Endostatin
PA: pulmonary artery
PAH: pulmonary hypertension
VEGF: vascular endothelial growth factor
