# Supplementary figures and images for: Increased Lung Expression of Anti-Angiogenic Factors in Down Syndrome: Potential Role in Abnormal Lung Vascular Growth and the Risk for Pulmonary Hypertension
Source: PLoS One. 2016 Aug 3;11(8):e0159005. doi: 10.1371/journal.pone.0159005 (PMC4972384; doi:10.1371/journal.pone.0159005)

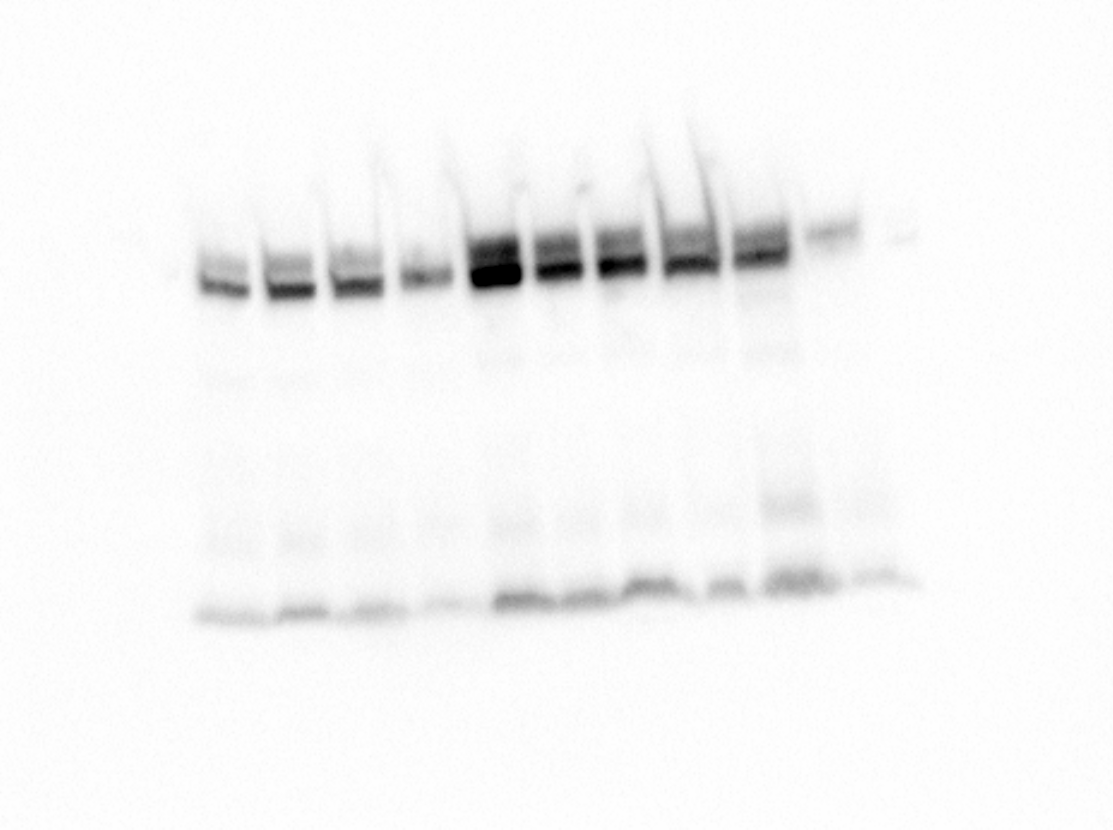

Supplement: S1 Fig — Western blot gel probed with amyloid protein precursor (APP). (TIF) [file pone.0159005.s001.tif]

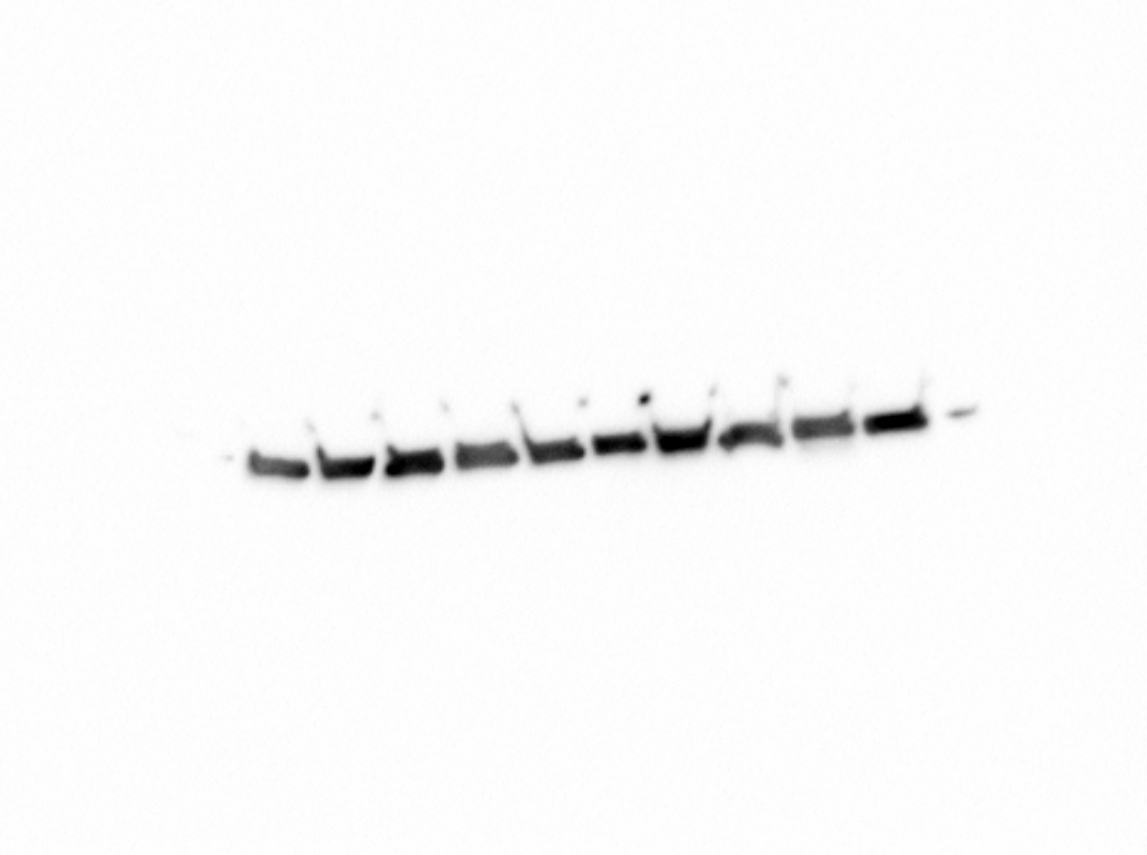

Supplement: S2 Fig — Western blot APP gel, probed with actin endogenous control. (TIF) [file pone.0159005.s002.tif]

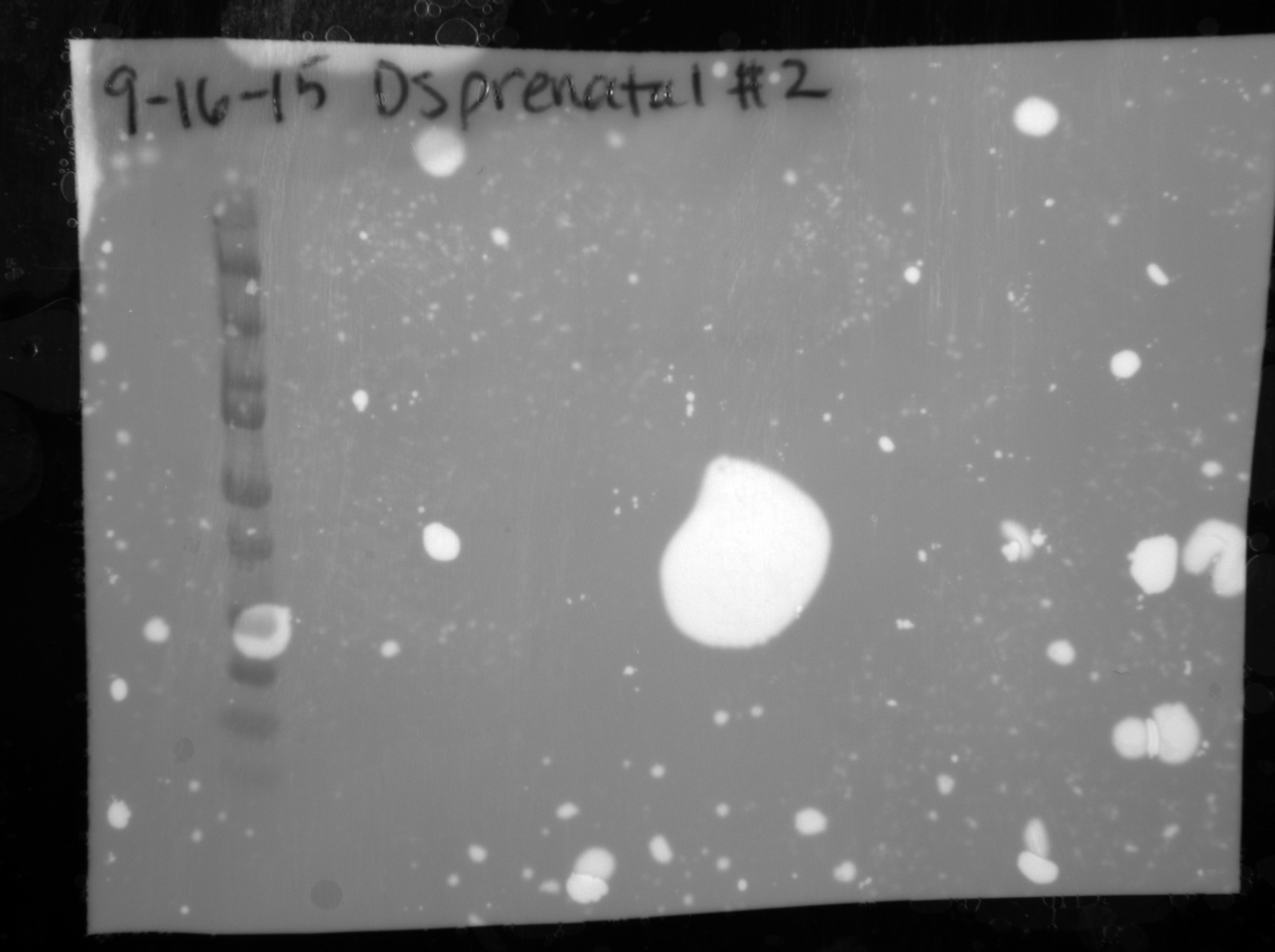

Supplement: S3 Fig — White light image of APP western blot. (TIF) [file pone.0159005.s003.tif]

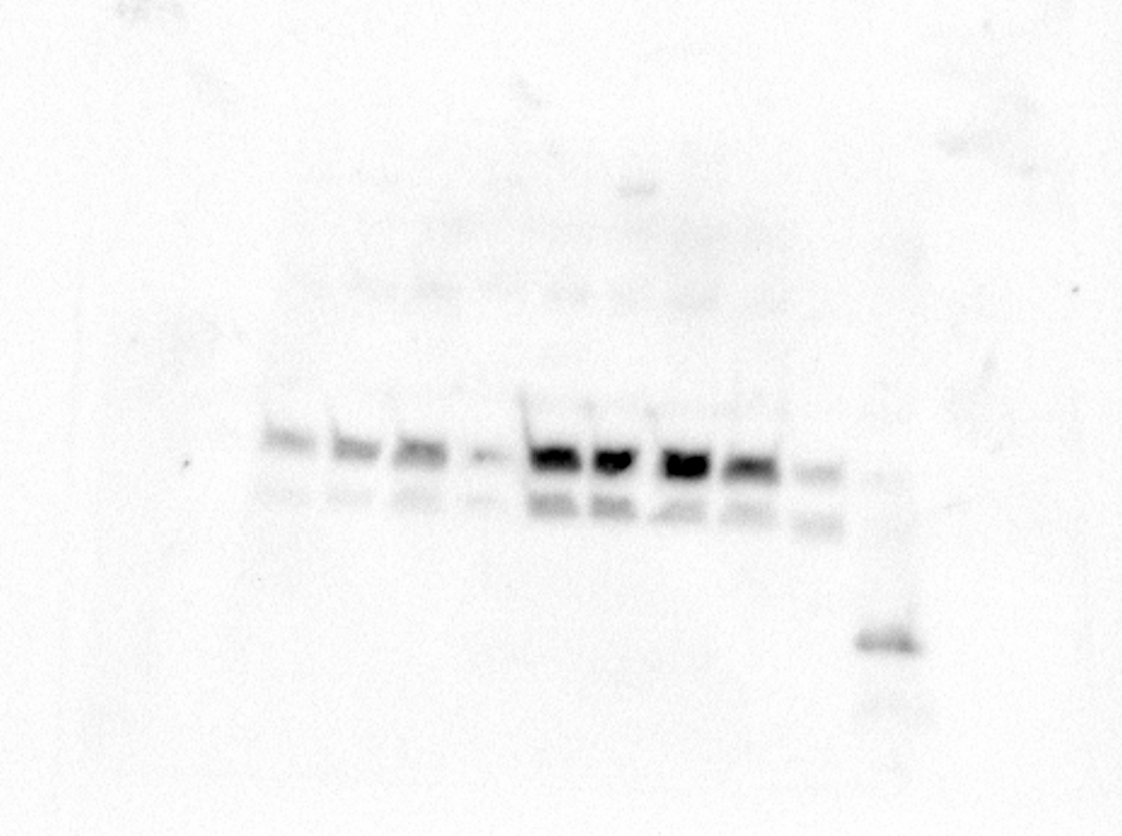

Supplement: S4 Fig — Western blot gel for Down syndrome critical region 1 (DSCR1). (TIF) [file pone.0159005.s004.tif]

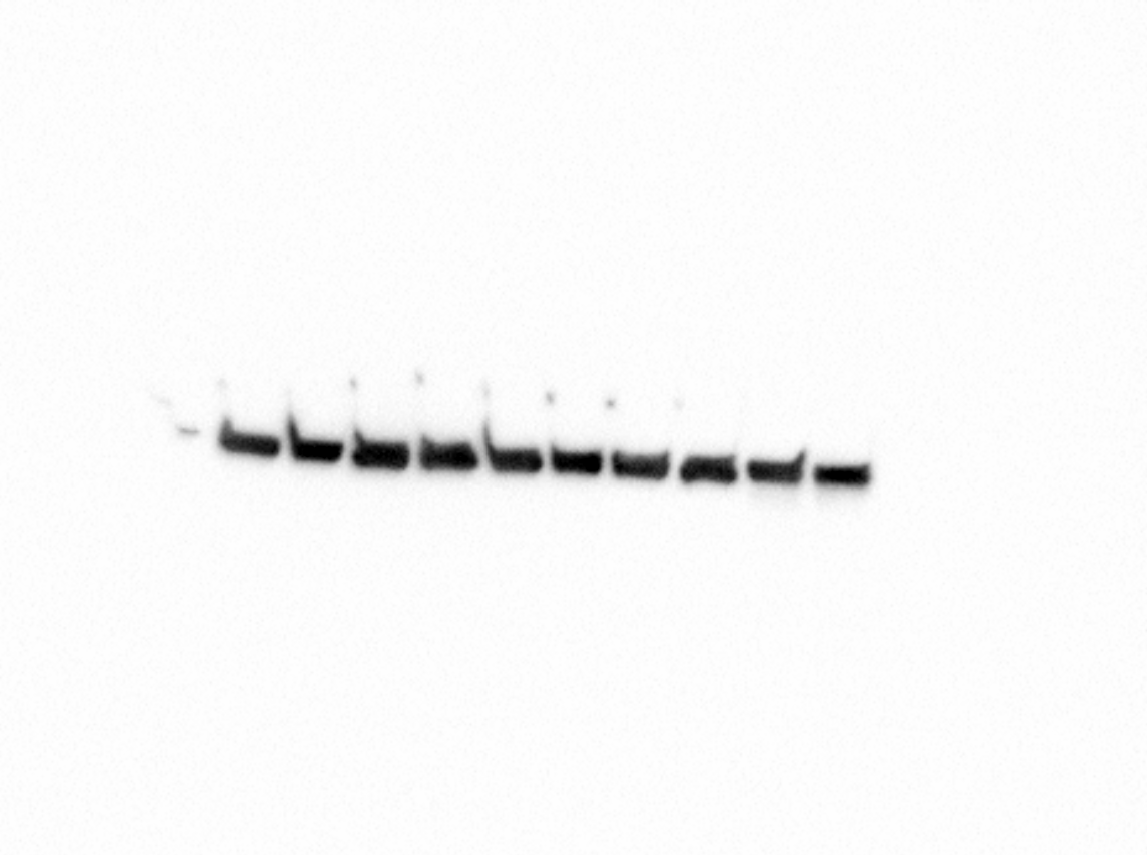

Supplement: S5 Fig — Western blot gel, actin control for DSCR1. (TIF) [file pone.0159005.s005.tif]

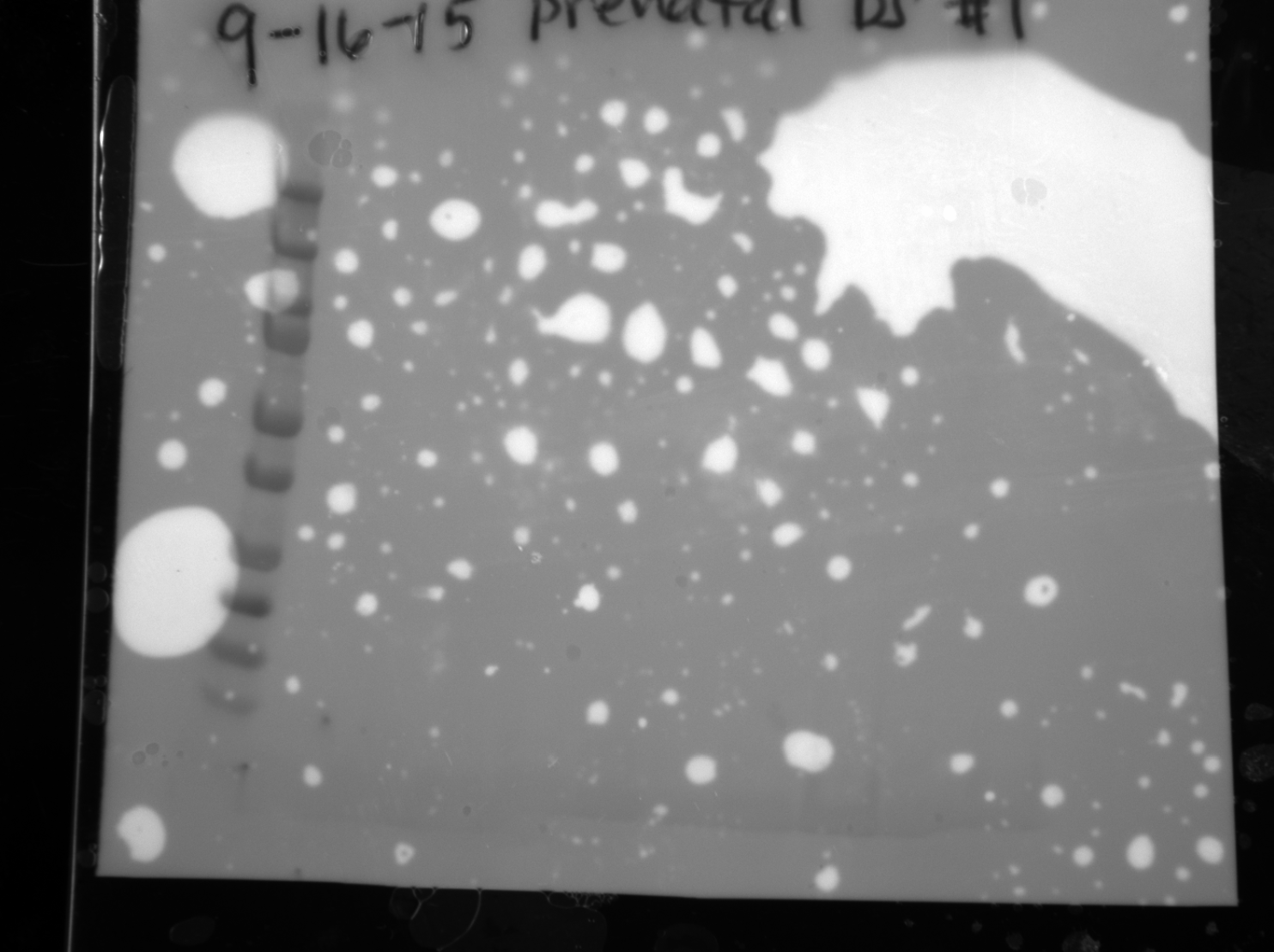

Supplement: S6 Fig — White light image of DSCR1 western blot. (TIF) [file pone.0159005.s006.tif]

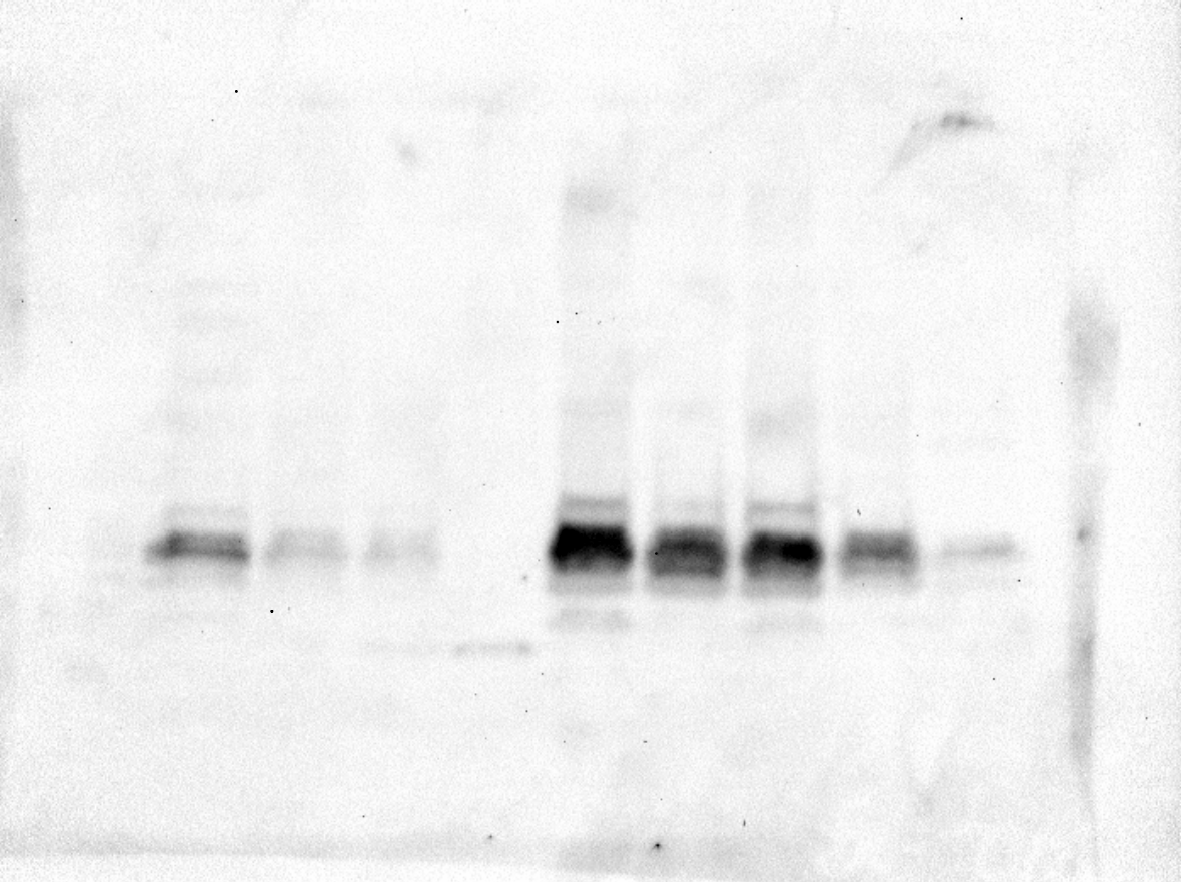

Supplement: S7 Fig — Western blot gel for endostatin (ES). (TIF) [file pone.0159005.s007.tif]

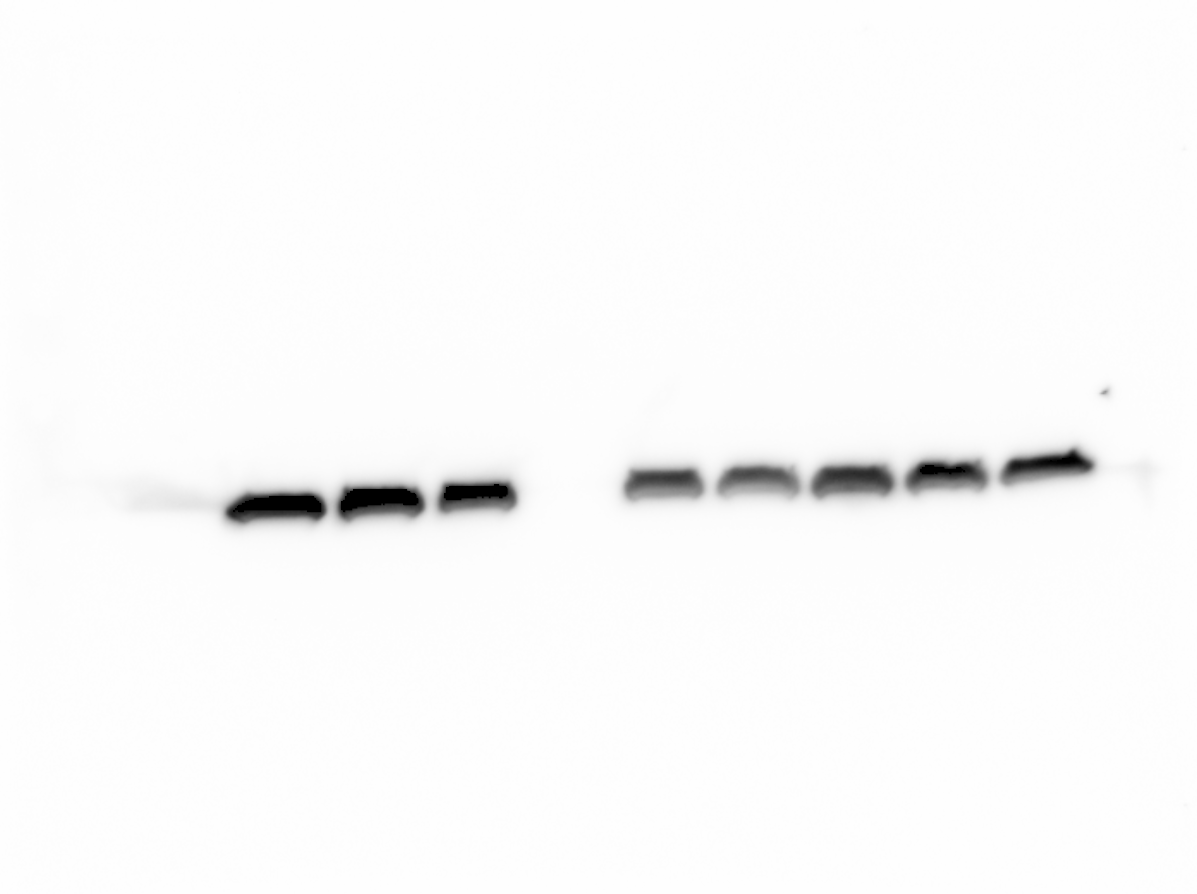

Supplement: S8 Fig — Western blot gel, actin control for ES. (TIF) [file pone.0159005.s008.tif]

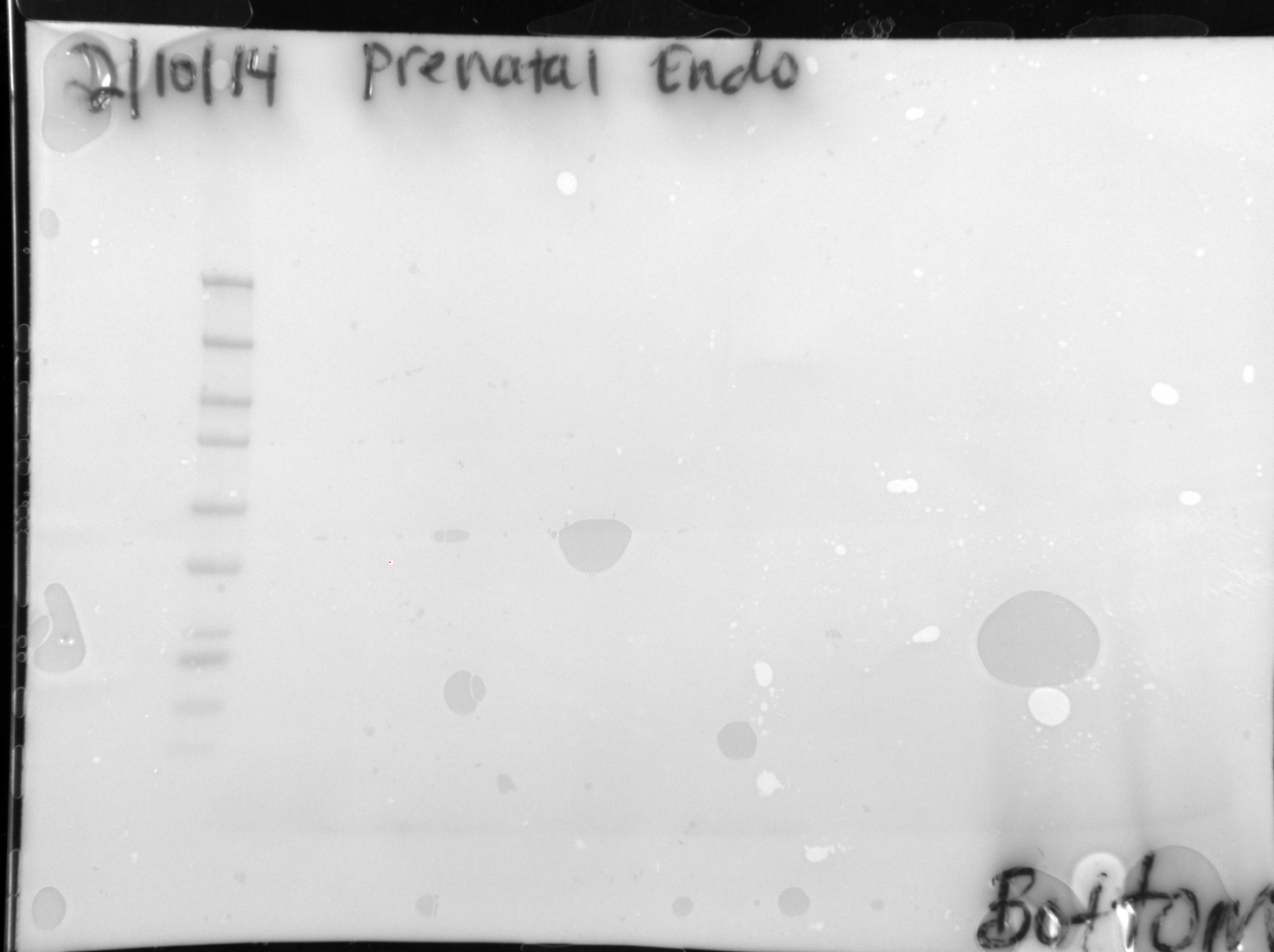

Supplement: S9 Fig — White light image of endostatin western blot. (TIF) [file pone.0159005.s009.tif]
